# Feasibility Studies on Si-Based Biosensors

**DOI:** 10.3390/s90503469

**Published:** 2009-05-11

**Authors:** Sebania Libertino, Venera Aiello, Antonino Scandurra, Marcella Renis, Fulvia Sinatra, Salvatore Lombardo

**Affiliations:** 1 CNR – IMM Catania, Italy; E-Mail: salvatore.lombardo@imm.cnr.it; 2 Università degli Studi di Catania, Dipartimento di Chimica Biologica, Chimica Medica e Biologia Molecolare, Catania, Italy; E-Mails: vera.aiello@imm.cnr.it; renis@unict.it; 3 Università degli Studi di Catania, Dipartimento di Scienze Biomediche, Catania, Italy; E-Mail: sinatra@unict.it; 4 Laboratorio Superfici e Interfasi (SUPERLAB), Consorzio Catania Ricerche, Catania, Italy; E-Mail: ascandura@unict.it

**Keywords:** Si-based biosensors, biological molecules immobilization, glutaraldehyde, glucose oxidase, DNA strands, metallothioneines, microelectronic compatibility

## Abstract

The aim of this paper is to summarize the efforts carried out so far in the fabrication of Si-based biosensors by a team of researchers in Catania, Italy. This work was born as a collaboration between the Catania section of the Microelectronic and Microsystem Institute (IMM) of the CNR, the Surfaces and Interfaces laboratory (SUPERLAB) of the Consorzio Catania Ricerche and two departments at the University of Catania: the Biomedical Science and the Biological Chemistry and Molecular Biology Departments. The first goal of our study was the definition and optimization of an immobilization protocol capable of bonding the biological sensing element on a Si-based surface via covalent chemical bonds. We chose SiO_2_ as the anchoring surface due to its biocompatibility and extensive presence in microelectronic devices. The immobilization protocol was tested and optimized, introducing a new step, oxide activation, using techniques compatible with microelectronic processing. The importance of the added step is described by the experimental results. We also tested different biological molecule concentrations in the immobilization solutions and the effects on the immobilized layer. Finally a MOS-like structure was designed and fabricated to test an electrical transduction mechanism. The results obtained so far and the possible evolution of the research field are described in this review paper.

## 1. Introduction

The ability to detect biomolecular interactions is of extreme importance in medical, pharmaceutical and biotechnological research and development. Biosensors have been developed for this purpose [1-3]. Their increasing importance in everyday life is driving a merger of the microelectronics and biomedical communities. The common objective is the production of devices ready for mass production that will perform accurate analyses.

A biosensor is a device that transforms biochemical information (presence and/or concentration of a specific analyte), into an analytically useful signal. It can be schematically represented as two basic components connected in series: a biological recognition system (bio-receptor, usually acting with interactions at supramolecular level) and a physical-chemical transducer. The system may be completed by a signal amplifier and a microelectronic circuit to elaborate the signal. Usually, the biosensor and the signal processing circuitry are not integrated. Different types of biologically sensitive materials can be applied as recognition elements. They can be enzymes, antibodies, antigens [4], proteins [5], nucleic acids [6,7] or even living biological systems (e.g., cells, plants, organs or whole organisms) [8].

Over the last 20 years there has been a growing interest in creating microbiosensors, fabricated in Si-compatible technologies, to be integrated within microelectronic circuits. The reason is that silicon-based devices would provide a lot of potential advantages such as small size and weight, fast response, high reliability, low output impedance, the possibility of automatic packaging at wafer level, on-chip integration and a signal processing scheme with the future prospect of low-cost mass production of portable microanalysis systems. In fact, among microelectronic materials, silicon (Si) has the most mature and low cost technology. Moreover, the Metal-Oxide-Semiconductor (MOS) system based on silicon as semiconductor and on SiO_2_ as dielectric is one of the key enabling technologies of these last fifty years. Complementary MOS (CMOS) technology has allowed the development of VLSI circuits with unprecedented performances at an exceptionally low cost for most of digital, analog, mixed signal, and RF circuits. The basis for this impressive progress is the exceptional quality of the Si/SiO_2_ system in terms of interface and bulk defects, low cost robustness to electrical and mechanical stress, scalability of the transistors, etc. Finally, SiO_2_ based matrixes have been proved to be a very useful support for the immobilization of biological molecules thanks to their capability of retaining biological activity. Many goals will be achieved using Si-based materials: *i*) the possibility to shrink the devices, implying reduced molecular diffusion path, faster kinetics and an improvement of the analytical performance of the device [9,10]; *ii*) the possibility to create micro-structured devices achieving complex functions, e.g. micro-total-analysis-systems; *iii*) the integration on the same chip of the electronics and/or photo-electronics needed for detection; *iv*) the possibility to make *in vivo* physiological monitoring; it implies lower reagent consumption, hence minimized sample volumes, lower energy consumption, and less space requirement (sensor portability). It should be mentioned that conventional biosensors need extensive packaging, complex electronic interfaces and regular maintenance or reactivation.

Finally, electrical sensing is considered one of the main goals to achieve in the next generation of biosensors since it could promote their integration within complex electrical circuits. Due to their simple principle of measurement and integrable signal processing on chip, biosensors that are based on electrochemical transducer principles are the most common sensor devices produced so far [11-20] and used in everyday life. Moreover, they are the most easily integrable in a microelectronic circuit, thus they would minimize the integration efforts in a complex circuitry.

The immobilization of the biological probe onto the transducer surface plays an important role in the overall performances of biosensors. Three main issues must be considered: *i*) the sensing surface must be biocompatible; *ii*) the immobilization protocol must not degrade the inorganic part of the sensor (for Si-based devices, VLSI compatibility); *iii*) the biological molecule must be anchored to the solid surface avoiding its denaturizing or the loss of its activity. The environment of the immobilized probes at the solid surface depends upon the mode of immobilization and can differ from that experienced in the bulk solution. These issues have been the target of many works in literature [10,21-26]. The most used approach is the formation of covalent bonds with the solid surface [10,24,25,27-29], often using bifunctional reagents to bridge the biological molecule and the functionalized sample surface. If silicon dioxide is used, the thermal processing used (wet or dry) may produce quite different results [23]. The immobilization procedure must be optimized to obtain the maximum surface coverage and to prevent the biological molecule denaturation and/or the loss of its specific property, e.g. for an enzyme its enzymatic activity [30,31].

Si-based biosensors, as well as conventional microelectronic devices, must be fully characterized using standard microelectronics techniques allowing biological molecule monitoring. In this way, the new technology costs are contained, since no new equipment is needed. Different techniques were used: X-ray Photoelectron Spectroscopy (XPS) and Atomic Force Microscopy (AFM). The first one provides information on chemical bonds and molecular composition of the material surfaces, combined with a high surface specificity; while the second one allows a careful topographic inspection of the surface. Finally, spectrophotometric techniques were also used. The above mentioned techniques were used to study the immobilization of three different biological molecules on SiO_2_ surfaces: glucose oxidase (GOx), DNA strands and metallothioneine (MT).

GOx is 160 kDa homodimeric globular protein, with a tightly bound (K_a_ = 1×10^-10^) flavin adenine dinucleotide (FAD) per monomer. The overall dimer dimensions measured by X-ray crystallography are 6.0×5.2×7.7 nm^3^ [32]. GOx, as all peptides and proteins, is a polymer of α-amino acids; it includes 580 amino acids, the FAD cofactor, six N-acetylglucosamine residues, three mannose residues and 152 solvent molecule acids [32]. As it is well known, the general chemical structure of an α-amino acid (excluding proline) is R-CH-NH_2_–(COOH). The enzyme catalyzes the oxidation of β-D-glucose to D-glucono-1,5-lactone by a reaction that can be summarized in two steps: i) glucose oxidation with the enzyme reduction, ii) re-oxidation of the enzyme with consumption of molecular oxide (O_2_) and production of hydrogen peroxide (H_2_O_2_) [33]. This enzyme is usually employed when the glucose concentration in the blood must be measured, hence GOx based micro-biosensors [34] would have immediate applications in monitoring diabetes [35].

The second biological molecule immobilized was single strand DNA (ssDNA). DNA molecules are charged macromolecules and the direct hybridisation event is an affinity binding process. DNA is a poly-anion with negative charges along its phosphate backbone. Double strand DNA can be considered as a circular cylinder (with a diameter of about 1.5–2 nm) with electrostatic charges evenly distributed about the cylindrical surface [36]; the length of a DNA probe depends on the number of nucleotides and the length of a nucleotide (or base) is about ∼ 0.34 nm [6]. To selectively recognise a unique human DNA sequence, DNA probes must be at least 16 bases long [37]. DNA recognition methods have assumed a primary importance in the genetic diseases' diagnosis.

Finally, MTs, extracted from rabbit liver, have a low molecular weight (<7,000 Da) [38] and ar cysteine-rich. They bind heavy metals such as silver (Ag), cadmium (Cd), zinc (Zn), copper (Cu) and mercury (Hg). Their dimensions, obtained using dark-field electron microscopy, are 3.6×2.5×1.6 nm^3^. The primary structure consists of 60-61 amino acid residues, 20 of which are cysteines, while there is a lack of aromatic and histidine residues. Not all the cysteines are involved in disulphide bonds and are able to link 7-14 metal ions per MT via thiol groups –SH [39]. This protein could be used as new sensitive element for the detection of heavy metal ions presence in liquid environment.

## 2. Results and Discussion

### 2.1. Immobilization protocol: definition and optimization

**Figure 1.**
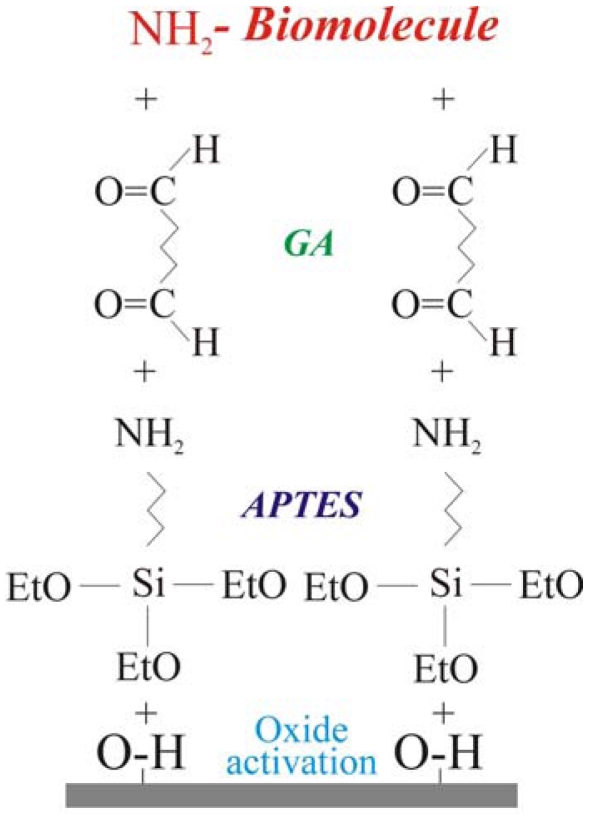
Schematic structure of the probe immobilization protocol. The four immobilization steps, oxide activation, silanization, linker molecule deposition and biological molecule immobilization are indicated as: oxide activation, APTES, GA, biomolecule (NH_2_ terminated), respectively.

The biological molecule immobilization procedure, reported schematically in Figure 1, requires four steps. The first one is oxide activation. It consists in the sample immersion in an ammonia and hydrogen peroxide water solution (SSC, ratio NH_2_:H_2_O_2_:H_2_O 1:1:10) at 70°C for 20 minutes. The process leaves OH terminated groups on the surface. This step is fundamental when the oxidation process is performed in dry ambient, in fact, the surface must be pre-treated before the second step (silanization) in order to maximize the -OH groups available to bond the silane groups [23]. The second step consisted of a treatment in vapours of 3-aminopropyltriethoxysilane NH_2_-(CH_2_)_3_-Si(OC_2_H_5_)_3_ (APTES) for 1 h. After silanization, the samples were cured under vacuum at 80 °C for 40 min. This process terminated the surface with -NH_2_ groups. The last two steps were linker molecule immobilization [surface terminated with –(C=O)H groups] and amino terminated biological molecule bonding. The linker molecule deposition was carried out using glutaraldehyde (GA), 2.5%, in phosphate buffer solution (PBS). GA is a linear molecule (CHO-(CH_2_)_3_-CHO) with one aldehydic group (CHO) at each end. Finally, the biological molecule immobilization was carried out for times ranging from 2 h up to 48 h at room temperature using water or PBS solutions depending on the molecule to immobilize (see experimental for details). The power of this method is that, once optimized, it may be used for any biological molecule having amino groups free for bonding. The details of the immobilization procedure for GOx [30,31,40], DNA [41] and MT [42] probes are fully reported elsewhere.

**Figure 2.**
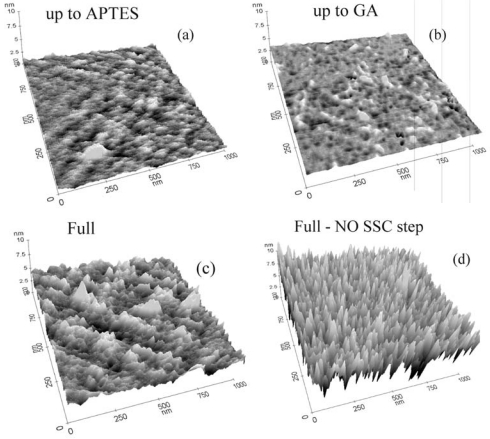
AFM images of: (a) up-to-APTES, (b) up-to-GA, (c) fully processed (with GOx), and (d) fully processed (with GOx) without the first step (NO SSC step). The vertical scale is 10 nm while the horizontal scales are both 1 μm. Higher peaks appear lighter on a gray scale.

The protocol effectiveness was tested using AFM and XPS measurements. In particular, the AFM analyses for all the intermediate steps of the immobilization process for GOx are reported in Figure 2. It should be stressed that the immobilization steps are the same for all the biomolecules reported in this work. Figure 2a shows the result on the sample that underwent oxide activation and silanization. The AFM measurements of this sample indicate a surface rms value of 0.22 nm. We used the same vertical scale in all three-dimensional images to allow a direct comparison of the samples. It should be noted that the APTES layer, if the surface is properly functionalized, has a physical thickness of 0.8±0.1 nm [43]. The AFM results suggested a good sample coverage. The highest peaks observed in Figure 2c are due to APTES polymerization. Samples that did not undergo oxide activation showed more polymerized sites (see ref. 40), indicating that a lower number of active sites was available for GA immobilization.

When the GA was deposited on the sample surface, only small modifications of the surface morphology occurred (Figure 2b). In fact, the surface rms, as measured by AFM, was 0.19 nm, barely distinguishable from the data extracted from the previous sample measurements. The data clearly indicated that the surface did not undergo relevant modifications, detectable by AFM. Measurements carried out on a sample that underwent the full immobilization process with GOx molecules are shown in Figure 2c. A clear modification of the surface occurred due to enzyme immobilization, with the appearance of peaks having the same height and good distribution on the sample surface. The rms value for this sample was 0.59 nm. Finally, the comparison of Figures 2c and d allowed us to make some considerations on the immobilization protocol, particularly on the importance of the first two immobilization steps: oxide activation and APTES functionalization. The data showed that oxide activation is fundamental for achieving the best surface coverage. If the sample did not undergo this step, the surface coverage was not uniform. The rms roughness of the sample not subjected to oxide activation (1.46 nm) was much higher than the rms roughness of the fully processed sample (0.59 nm). This indicated that surface coverage was not uniform and the peak heights were not all the same, indicating that there were regions where, probably, the GOx did not bind to the surface. Our results demonstrated that the oxide activation had a double effect: it reduced the surface contaminations and allowed the formation of more surface sites available for silanization (OH terminations).

**Figure 3.**
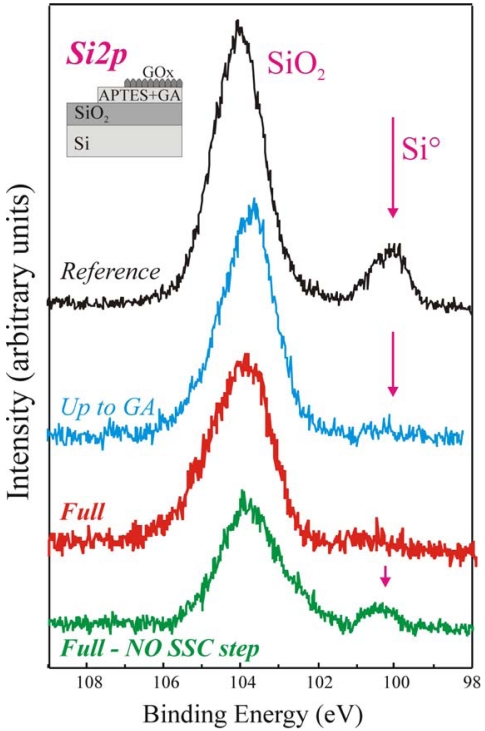
High statistic acquisition mode XPS Si2*p* spectral regions of the reference (black line), up to GA (blue line), fully processed with SSC (GOx molecule, red line, Full) and fully process without SSC (green line, Full-NO SSC step) samples. A schematic of the immobilization steps is also reported (top left).

Similar conclusions can be drawn measuring the samples by XPS. In particular, the Si 2*p* peaks of the same samples, shown in Figure 3, provide interesting information. The reference sample (in black) exhibited two components having binding energies of 99.7 eV and 104 eV, assigned to Si° and SiO_2_ respectively [44,45]. The SiO_2_ component is centred at 104 eV instead of 103.4 eV, as expected, due to differential charging between the SiO_2_ layer and the Si substrate [45]. The Si° component is visible since the oxide thickness is small (6.5 nm) and the substrate signal succeeds to pass the oxide and reach the detector. Our results [40-42] demonstrate that if the organic film deposited on the sample surface is uniform the substrate Si° signal is fully shield. In fact, the up-to-GA sample, processed using our protocol, does not exhibit such signal (blue line). The same result is, clearly, obtained for the Full+SSC sample (red spectrum), since a further layer is deposited on the surface (see schematic). The situation is completely different for the Full-NO SSC step sample (green spectrum in Figure 3). Even if a thick layer is deposited on the oxide it still exhibits the Si° component.

This result clearly indicated that the immobilization procedure did not produce a uniform film for the full-NO SSC step sample, despite of the fact that the enzyme was correctly immobilized. The results confirmed that the immobilization procedure without oxide activation was not the best achievable on our SiO_2_ surfaces [40,41,46]. The great improvement in film uniformity measured on the samples that underwent Full+SSC protocol can be explained considering that the oxide activation, we introduced as the first step, allowed us to increase the number of available sites for enzyme bonding and to obtain a final uniform deposition. The enzymatic activity measurements confirmed these results (see after) [46].

It should be mentioned that a correct silanization procedure is fundamental for the uniform coverage of the surface. We demonstrated that if silanization is not properly carried out, APTES polymerization occurs as immediately observed by AFM measurements [40].

**Figure 4.**
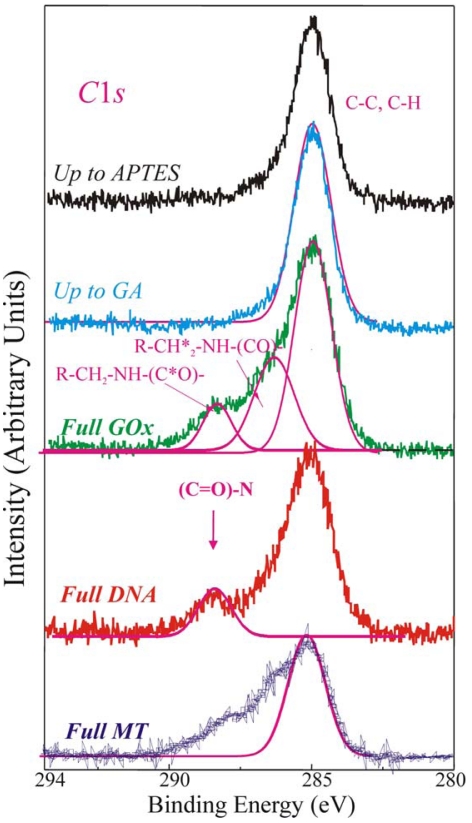
High statistic acquisition mode XPS C1*s* spectral regions of: up-to-APTES (black line), up to GA (light blue line), fully processed with GOx (green line, Full GOx), fully processed with DNA (red line, Full DNA) and fully processed with MT (blue line, Full MT) samples. The magenta lines are the simulated peaks.

The direct evidence of the biomolecules immobilization on the sample surface was provided by the analysis of the XPS C1*s* spectra reported in Figure 4, of a sample stopped after the APTES step (up to APTES, black line), after the GA (up to GA, light blue line), fully processed with GOx (green line, Full GOx), fully processed with DNA (red line, Full DNA) and fully processed with MT (blue line, Full MT) samples.

The C 1*s* component, centred at 284.8–285eV and clearly observed in the first two samples (up to APTES and up to GA), is assigned to C-C and C-H bonds. The magenta line superimposed to the experimental spectrum was a simulation of the C-C and C-H XPS peaks. The up-to-GA sample showed only this peak, and an additional weak shoulder at about 287 eV attributed to the R-CHO groups of GA. It should be reminded (see introduction) that the GA is a linear molecule (CHO-(CH_2_)_3_-CHO) with one aldehydic group (CHO) at each end. The other samples showed other components at higher binding energies.

The Full GOx samples exhibited at least other two components at 286.3–286.5eV, assigned to R-CH_2_*-NH-(CO)-, and at 288.3eV, assigned to R-CH_2_-NH-(C*O)-chemical groups respectively. The magenta lines in Figure 4 are the simulated peaks superimposed to the experimental data to allow one an easier identification of the different peaks. They are characteristic of the proteins and expected if the enzyme is deposited on the sample [31,40,42,44-46].

When DNA strands are immobilized on the sample (Full DNA), besides of the C1*s* peak at 285 eV, the XPS spectrum exhibited a component at about 288 eV. It has been assigned to –(C=O)-N chemical groups. They are characteristic of the DNA nitrogen bases and their presence is expected only when the DNA is deposited on the sample [41,42,44,45], as demonstrated by their absence in the other spectra compared in the same Figure. These results confirmed the DNA presence on the sample, in fact the component of C1*s* at 288 eV we assigned to the nitrogen bases of DNA is specific of DNA presence in our samples.

**Figure 5.**
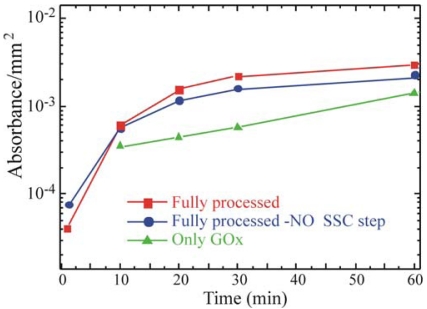
Glucose oxidase activity as a function of time for: bulk SiO_2_ samples Fully processed (red squares), fully processed without SSC (blue circles, -NO SSC step), with only GOx (green triangles).

Finally, the Full MT sample shows at least other two additional components at 286.3 eV assigned to R–CH_2_*–NH–(CO)–, and 288.3 eV, assigned to R–CH_2_–NH–(C*O)– chemical groups, characteristic of the proteins, as already observed for the Full GOx sample. In order to understand if the immobilization procedure has affected the bio-molecules' characteristics, the enzymatic activity of the fully processed GOx samples was monitored using a simple spectrophotometric assay [46]. GOx activity was monitored on the Full+SSC sample, on the Full-NO SSC step sample and on the sample that underwent only GOx deposition without previous surface functionalization (only GOx). The results are summarized in Figure 5. All data were normalized to the real area of the sample.

They show an increase in the enzymatic activity when the oxide activation is carried out before silanization (red squares) with respect to the Full-NO SSC step sample (blue circles). The only-GOx sample (green triangles) exhibited an enzymatic activity lower than the one measured for both the samples that underwent chemical immobilization. The comparison of the absorbance values with those obtained from the free enzymes in solution, allowed us to estimate a concentration of active GOx on the SiO_2_ samples of about 0.002 U mL^-1^.

**Figure 6.**
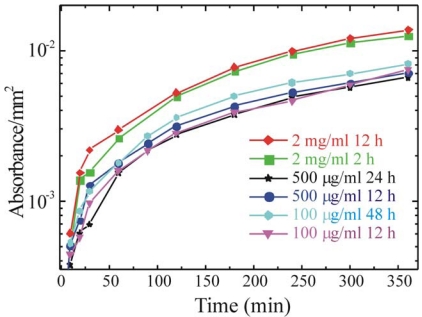
Glucose oxidase activity as a function of time for samples immersed in: 2 mg/mL for 12 h (red rhombuses) or 2 h (green squares); 500 μg/mL for 24 h (black stars) or 12 h (dark blue circles); 100 μg/mL for 48 h (light blue symbols) or 12 h (magenta triangles).

In order to optimize the immobilization protocol also the immobilization step parameters must be studied. In particular, we tested solutions differing for the enzyme concentration in a range from 100 μg/mL up to 2 mg/mL and immersion times ranging from 2 h to 48 h. These parameters have a great importance when a large scale production is performed. In fact, to immobilize the enzyme on large surfaces, larger amounts of solution must be prepared. Moreover, immobilization for times longer than needed will causes a reduction in the production rate. The results, obtained measuring the enzymatic activity are summarized in Figure 6.

The data clearly show that similar absorbance values are obtained regardless of the immobilization time for solutions of 500 μg/mL and 100 μg/mL, while more than a factor two increase is obtained for 2mg/mL solutions. For this concentration is interesting to observe that there is no difference for immersion times of 2 and 12 h, thus suggesting that a strong reduction in the immobilization time can be obtained.

**Figure 7.**
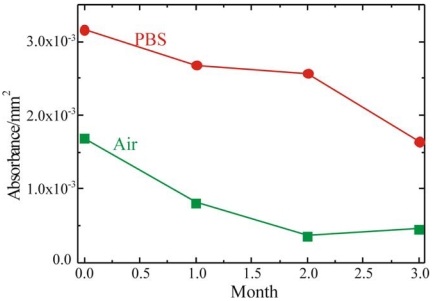
Glucose oxidase activity as a function of storage time for bulk SiO_2_ samples stored in PBS at 4°C (red dots) and in air at RT (green squares).

To fabricate a sensing layer using GOx as sensitive element, it is fundamental to understand the device shelf life and to determine the best storage conditions. To this purpose the enzymatic activity of the fully processed sample, stored either in buffer solution (PBS, 0.1M pH 6.5) at 4°C or in air at RT, was monitored. The results of these measurements are summarized in Figure 7 where the red circles represent the sample stored in PBS, while the green squares represent the sample stored in air. Both samples were monitored immediately after immobilization and after 1, 2 and 3 months. The absorbance values reported in Figure were detected 60 min after the reaction started. The samples stored in PBS retained their activity over longer periods of time. On the other hand, the samples stored in air showed an immediate decrease in the enzymatic activity, already after the first month of aging.

Another important test is the dependence of the layer efficiency on the SiO_2_ surface characteristics. To this purpose, we thermally grow a thin Si layer (see Section 3 for the fabrication details) on various Si substrates having different doping and impurity concentrations and on quartz. The results, not shown, indicate that the surface immobilization efficiency is not affected by the doping and impurity concentration of the Si substrate.

A modified surface can be used to improve the device performances (in particular its sensitivity). As an example porous silicon dioxide can be fabricated. Measurements we performed on porous SiO_2_ fully processed samples demonstrate the enzyme presence within the pores and an enhanced enzymatic activity, due to the higher surface/volume ratio. An increase in sensitivity of one order of magnitude was obtained using a 3 μm thick layer of porous SiO_2_. The results are reported in [46]. A further improvement in the device sensitivity can be obtained by micropatterning of the surface. In particular, we demonstrated that a strong increase in the enzymatic activity was observed by ink-jet printing the GOx on a planar SiO_2_ surface [47]. The result so far reported indicated that the final device performances will strongly depend on the fabrication method and on the final device structure, hence it is not possible to infer the final device sensitivity only from the results here reported.

Finally, to check that both DNA strands and MTs keep their characteristics once immobilized, different measurements were performed. DNA single strands were tested electrically monitoring their ability to hybridize the complementary strands (see later).

**Figure 8.**
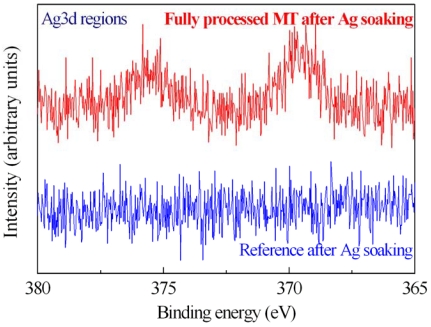
XPS spectra of Ag 3d regions for the samples: full MT (500 μg/mL, red line) reference (blue line) after Ag soaking.

The MT activity on immobilized samples was tested by measuring with XPS the spectra of fully processed samples after Ag salts soaking (Figure 8). The XPS characterization clearly shows the presence of Ag on the sample surface after immersion. In fact, the Ag 3d spectrum of one of them (500 μg/mL) is shown in Figure 8 (red line). The reference sample (blue line) doesn't exhibit traces of Ag after immersion in the same solution. This result indicated that the proteins maintained their ability in getter heavy metals also after the immobilization process. A careful description of the method and the results is provided in [42].

**Figure 9.**
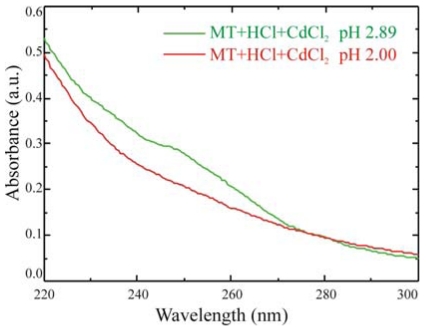
Absorbance spectra of a 100 mg/mL MT solution at different pH: 2.89 (green line) and 2.00 (red line).

Also spectrophotometric results can provide information on immobilized MT activity, as demonstrated in Figure 9, where the comparison between two solutions of 100 μg of MT/ml at different pH is reported.

At pH above 2.5, MTs getter Cd and a shoulder in the absorbance spectrum at 250 nm is clearly visible. When the pH is reduced below the 2.5 threshold, by adding hydrochloric acid, MTs release the metallic ions captured and the shoulder disappears. Experiments are in progress, on quartz samples, to test the mechanism on immobilized proteins.

### 2.2. Electrical Testing

As mentioned in the introduction, the possibility of obtaining an electrical signal as a result of the transduction mechanism can greatly expand the miniaturization potentiality of biosensors. In this perspective electrolyte-insulator-semiconductor (EIS) structures are quite intriguing. Their working principle is straightforward: the recognition event, occurring at the insulator/electrolyte interface, causes a change in the charge (potential) at that interface and it can, in principle, be detected. The capacitance in a EIS structure can be described as the built-up of several capacitors in series. The total capacitance (C*_tot_*) is the series of: the semiconductor capacitor (C_Si_); the insulator capacitor (C_ox_) and the capacitor provided by the layer given by the sensitive element (C_bio_) [19]. The recognition event will produce a change in the capacitance of C_bio_, that will produce also a change in the surface potential. Hence, a shift in the flat band voltage (V_FB_), associated to the flat band capacitance (C_FB_), may be detected.

**Figure 10.**
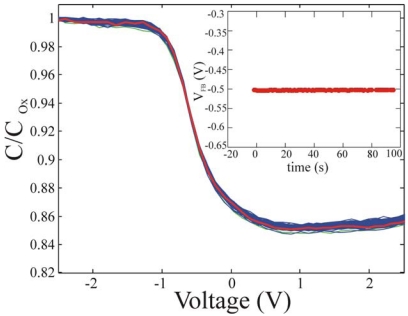
Capacitance voltage measurements carried out on reference sample. The first measure is reported in green cross. In the inset, the V_FB_ extracted from the CV curves as a function of time (aging).

The MOS-like devices were prepared as described in the experimental section, in which all the fabrication details are reported, and the immobilization procedure already described was used. After immobilization, the samples were dried under a gentle Nitrogen flow and measured using capacitance-voltage (CV) measurements at 1 MHz, with a repetition frequency of 10 Hz and the curves were acquired during 100s of measurements. The results are shown in Figure 10 for the reference sample.

The measurement reproducibility was tested on each set of measurements and on at least three samples per type. The data reproducibility and the absence of oxide aging were tested. If oxide aging was present, a shift in the flat band voltage (V_FB_) had to be observed as a function of time, having a direction depending on the trapped charges. The V_FB_ values as a function of time, as extracted from C-V measurements, and reported in the inset of Figure 10, did not change during measurements, indicating that no oxide aging was present [41,48]. For all measurements, the capacitance was normalized to C_ox_, measured in the device accumulation region (negative biases). A constant value of -0.50±0.05 V was detected for the V_FB_ of the reference samples and used for the comparison with the immobilized samples.

**Figure 11.**
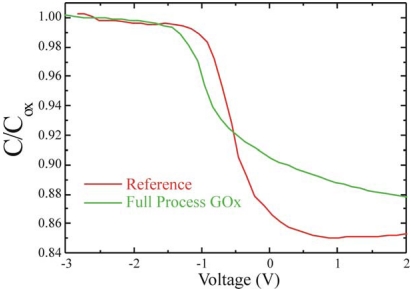
C-V measurements of the reference sample (red line) and of a GOx immobilized sample (green line).

Once verified the measurement reproducibility, GOx immobilized samples were tested. Also in this case no dielectric (C_bio_) aging was detected, but a clear shift of V_FB_ towards negative biases was monitored, as shown in Figure 11, where the C-V of both the reference (green line) and the GOx fully immobilized (green line) samples are compared. The V_FB_ approaches to -1.07±0.04 V (average value), hence, a shift of 0.57 V, well above the measurements indetermination (±0.05 V), was detected. This result confirmed that GOx immobilized on the dielectric surface introduced a positive charge as already observed by Wang *et al.* [49]. The most important conclusion that can be drawn from these data is that this device is very sensitive to the presence of organic layers, hence it is a promising candidate for the fabrication of fully electric (MOS-based) glucose sensors.

**Figure 12.**
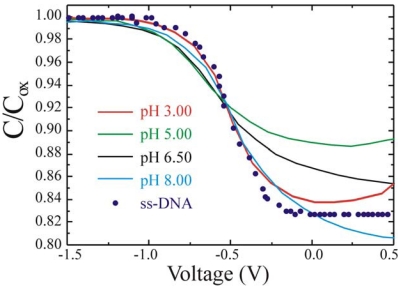
Normalized Capacitance Voltage curves of the reference sample as a function of the electrolyte pH (3.0 red line, 5.0 green line, 6.5 black line, 8 light blue line) and in presence of complementary ssDNA at pH of 6.5 (dark blue circles).

The V_FB_ behavior when ssDNA is immobilized on the SiO_2_ surface is quite different. Two different set of measurements were carried out to discriminate the different effects. In a first set of measurements the dependence of measurement from the solution parameters was tested. In fact, the solution parameters, pH and ionic concentration, can strongly affect the measurement, since they may give a contribution to the value of C_bio_, thus reducing the sensor performances [19]. C-V measurements were carried out on the reference samples as a function of the solution pH. An example is shown in Figure 12. The curves perfectly overlap (within the experimental errors) regardless of the buffer solution pH, from 3.0 (red line) up to pH 8 (light blue line). It should be mentioned we tested solutions ranging from pH 3 to pH 9 (data are not shown) and obtained the same results. Please note that the horizontal scale was expanded in the -0.5 region to better show the curves overlap.

It is known that SiO_2_ exhibits a pH dependence of ∼ 30 mV/pH in the observed pH range. Since no shift was observed, within the experimental errors, in the measured curves it is reasonable to assume that the SiO_2_ surface was perfectly passivated. In fact, it is known that the pH sensitivity depends on the density of OH groups at the SiO_2_/solution surface. The oxidation process employed to prepare the sample used in this work was in a dry ambient, hence the number of OH^-^ groups is strongly reduced. Moreover, since no permanent V_FB_ shift was observed as a function of time, also stressing the samples we may conclude that there was no dielectric aging during the measurements [41,48]. Finally, a PBS solution with the complementary oligonucleotide was spotted on the reference sample and the C-V measured to verify if the presence of the additional negative charge in solution cold cause a V_FB_ shift. The data, also shown in Figure 12 as circles, demonstrate that the C-V was not affected by the various pH or the DNA presence in the PBS solution.

**Figure 13.**
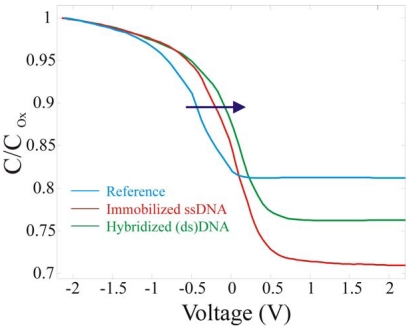
Normalized Capacitance Voltage curves of the reference sample (light blue line), fully processed ssDNA (red line) and of the hybridized dsDNA (green line). The arrow underlines the direction of the V_FB_ shift.

The second set of measurement we performed was aimed to verify if an electrical transduction could be achieved from DNA hybridization. The reference and the two fully processed samples were electrically characterized and the results are compared in Figure 13. The first test was carried out on reference samples (SiO_2_/Si, light blue line) and a V_FB_ constant value of -0.41±0.03 V was detected during the entire measurement time. Since there was no oxide aging, any shift in V_FB_ eventually detected must be attributed to the organic layer deposited on the oxide surface. Quite different is the V_FB_ behaviour when ssDNA is immobilized on the SiO_2_ surface. The fully processed ssDNA sample (red line) and the same sample after the hybridization process (green line) exhibited a clear shift in the V_FB_ values. The data show that V_FB_ approached to +0.06±0.01 V after the device full processing. DNA molecules have negative charges derived from the phosphate groups in aqueous solutions, hence a negative charge was trapped on the sample surface, as expected. The negative charge presence on the oxide caused positive V_FB_ shift, with respect to the reference sample, as already observed using different structures [14]. In particular, a +0.47±0.04 V shift on V_FB_ was detected.

The DNA immobilization causes a visible difference in the C_Min_ value. The reduction in the C_Min_ value (C^DNA^) to 0.7 of the C_ox_ can be attributed to the presence of the extra layer on the SiO_2_ surface. Knowing that C*_tot_* is the series of C_Si_, C_ox_ and C_bio_ and that C^ref^/C_ox_ is 0.8 while C^DNA^/C_ox_ is 0.7, with simple calculations the biological layer thickness can be evaluated, assuming a dielectric constant for the biological layer of 3 [12]. A value of ∼5.6 nm was obtained, in good agreement with the ssDNA length (6.8 nm). The final set of measurements of Figure 13 was aimed to demonstrate the device sensitivity to DNA hybridization. The hybridization caused a further shift of 0.07±0.02 V, always in the same direction, since more negative charges are immobilized on the surface. The V_FB_ approached to 0.13±0.01 V. The results are shown as a solid line. The shifts from the reference did not linearly increase, in fact the hybridized sample did not exhibit a shift double with respect to the ssDNA as one could assume at the first glance. This could be due to two effects: not all the DNA hybridizes and the well known counter-ion effect [20] takes place.

## 3. Experimental Section

### 3.1. Sample preparation

Si oxide thin layers were grown on 6 inch p-type CZ Si wafers (∼2 Ω×cm), n-type CZ and epitaxial Si wafers (∼2 Ω×cm), p^+^-type CZ Si wafers (∼0.001 Ω×cm), using two different processes. Samples to test the immobilization protocol were prepared by thermal oxidation in an O_2_ environment at 900°C for 30 min. The oxidation time was chosen to grow a thin oxide layer, 6.5 nm thick, as measured by XPS [31,40]. Samples to be used as MOS like structures were prepared by thermal oxidation at 950°C for 30 min, to grow 15 nm thick oxide. The final MOS-like structure is given by a thick CZ Si layer (∼ 500 μm) B doped to a concentration of ∼ 1.5×10^15^ B/cm^3^. The SiO_2_ layer was 15 nm thick, as measured by TEM microscopy (not shown). The upper contact was provided by a Phosphate buffer solution (PBS) drop of 1.5 μl. The estimated device area was ∼ 0.78 mm^2^ (a circle with 1 mm of diameter).

The biological molecule immobilization procedure consisted of four steps: (1) oxide activation; (2) silanization using 3-aminopropyltriethoxysilane (APTES); (3) linker molecule (GA) deposition and (4) biological molecule coupling. The solutions used were: 2 mg/mL GOx in PBS, 500 μg/mL MT in PBS, 100 μM ssDNA probe and 100 μM complimentary DNA strand (ssDNA-c).

DNA strand used were 20 bases long functionalized at the 5′ end with a NH_2_-C_6_ group. The sequence used was ATGCATGCATCGTACGTACG. DNA hybridization was monitored utilizing its complementary strand (ssDNA-c), having the sequence TACGTACGTAGCATGCATGC.

Samples for electrical measurements were scratched on the back with a diamond probe and a drop of silver paint was used to assure the electrical back contact.

### 3.2. Enzymatic activity measurements

GOx activity was determined by measuring the amount of H_2_O_2_ formed, using a commercial spectrophotometric glucose assay kit, purchased from Megazyme^®^. GOx enzyme catalyzes the oxidation of glucose to D-gluconic acid producing hydrogen peroxide (see introduction). In the presence of peroxidase (POD), hydrogen peroxide participates in a reaction involving p-hydroxybenzoic acid and 4-aminoantipyrine (both were provided in the kit) and a quinoneimine dye complex is formed, which is measured at 510 nm. GOx was diluted in a homemade PBS buffer solution. To test the activity of the enzyme immobilized on the SiO_2_ samples, they were inserted in cuvettes containing the above mentioned peroxide solution.

### 3.3. Measurement equipment

AFM measurements were performed in air at room temperature using an XE 150 by PSIA scanning probe microscope operated in the noncontact mode. AFM scans (typically 1×1 μm) were carried out on several surface positions to check the surface uniformity.

X-ray Photoelectron Spectroscopy (XPS) analyses were carried out using a Kratos AXIS-HS spectrometer. In the present study the Mg Kα_1,2_ radiation of 1253.6eV was used at 10mA and 15keV and at pass energy of 40eV of the energy analyzer. High statistics acquisition mode was done at step energy of 0.025 eV and 300 ms of acquisition time.

Absorbance measurements were carried out at 510 nm using a Varian Cary 50 spectrophotometer. GOx standard from Megazyme^®^ and GOx in a homemade dilution buffer (see before) were used to prepare the calibration curve. Absorbance was measured immediately after the reaction started and then every 10 min up to 4 hours. To monitor the immobilized GOx enzymatic activity, functionalized silicon samples (both bulk and porous SiO_2_) were placed in cuvettes containing both POD and D-glucose solutions. A cuvette containing only MilliQ water and a cuvette containing an unprocessed Si sample were used as references of GOx solution and of solid samples respectively, and measured using the same procedure described above. All measurements were carried out at room temperature. The absorbance values were normalized to the sample area.

Electrical measurements were carried out at RT using drops of PBS buffer solutions to provide the upper contact of the MOS-like structure having the SiO_2_+organic layer as dielectric. The electrical signal was generated by a Tektronics AWG2005 wave generator, measured using a BOONTON capacitance meter (1 MHz small signal), and collected by a TDS520B oscilloscope (2.5 kS/s, high resolution). All data were finally collected by a PC using an home made program implemented using the Labview^©^ software. Measurements were repeated to a frequency of 10 Hz in dark, to avoid photo induced carried generation. The bias ranged from -3V to +3V. Each set of measurements (consisting in 120 CV curves) was performed triplicate and the V_FB_ reported are the average on all the measurements.

### 3.4. Reagents

3-Aminopropyltriethoxysilane (APTES), glutaraldehyde solution (GA, grade II), glucose oxidase (type X-S, Aspergillus niger, 179,000 U g^-1^ solid, Sigma), Metallothionein from rabbit liver were purchased from Sigma Chemical Co., St Louis (USA). Amino modified single-stranded DNA (ssDNA) had 20 bases and a NH_2_-C_6_ group attached at the 5′ end. Its sequence was ATGCATGCATCGTACGTACG, while its complementary (ssDNA-c), had the sequence TACGTACGTAGCATGCATGC. Both were purchased by Operon Biotechnologies GmbH. Silver paint was purchased from Touzard & Matignon (France). The other chemicals used were purchased from Carlo Erba Reagenti (Italy). A commercial glucose assay kit was purchased from Megazyme^®^. Deionized, MilliQ water, having 18 MΩ resistivity, was used.

## 4. Conclusions

We optimized a protocol to covalently immobilize biological molecules having a free amino terminated group on SiO_2_ surfaces. The protocol versatility was tested immobilizing the enzyme glucose oxidase, amino-terminated DNA strands and the protein metallotyoneine. The protocol we used consisted in four steps: (1) oxide activation; (2) silanization using 3-aminopropyltriethoxysilane (APTES); (3) linker molecule (GA) deposition and (4) biological molecule coupling. The first was introduced to improve the immobilization quality. We determined the biological layer presence and uniformity by AFM and XPS measurements. In particular, the biological molecule presence was univocally determined monitoring the XPS C 1*s* signal in the rang 280-294 eV.

From the enzymatic activity data carried out on GOx fully processed samples we concluded that the immobilization procedure on SiO_2_ bulk supports did not denature or destabilize the enzyme that the samples must be always stored in PBS at 4°C so that their activity can be retained for months.

The electrical characterization of MOS-like capacitors fully processed with GOx exhibited a negative shift (∼ 0.6 V), typical of positive charges presence, of the V_FB_, indicating the device sensitivity to the enzyme presence. The MOS-like structures tested are also sensitive to DNA immobilization and hybridization, as demonstrated by a positive shift in the V_FB_ of +0.47±0.04 V after immobilization and by a further +0.07±0.02 V shift when hybridization occurs.

Finally, to verify that immobilized MTs were still able to link the heavy metals, we used the XPS technique. The measurements show the presence of Ag only in the MT immobilized sample that underwent at Ag soaking.

Preliminary results on GOx immobilized samples suggest that the sensor sensitivity will depend on the fabrication characteristics. In fact, we found that the enzymatic activity increased when the immobilization layer was given by porous, instead of planar, SiO_2_. Also the immobilization method, immersion or ink-jet printing strongly modifies the enzymatic activity results, thus suggesting different sensor sensitivity, while the substrate characteristics (doping and impurity content) do not affect the final activity. Hence it is not possible to infer the final device sensitivity only from the results reported in this paper. Further experiments are in progress to answer this interesting question.
